# Prof. F. H. Hamilton, M. D., and the N. Y. City Commissioners of Public Charities and Correction

**Published:** 1874-06

**Authors:** Frank H. Hamilton

**Affiliations:** Surgeon-in-Chef, Reception Hospitals


Prof. F. H. Hamilton, M. D., and the New York City Commissioners of Public Charities and Correction.—Commissioner Stern in a communicaiion to the Board of Commissioners of Public Charities and Correction, on April 20th, made certain statements derogatory to Prof. Hamilton. In a letter addressed to the Board, Prof. Hamilton replies as follows:

*To the Commissioners of Public Charities and Correction:*

Gentlemen:—My attention has just been called to a communication made to your Board, April 20, by one of its members, Commissioner Stern, and published in the *City Record* of April 27, and also in the *Evening Post*, relating to myself and the Reception Hospitals, which communication contains errors of statement as to the facts of sufficient importance to demand correction. Mr. Stern says, speaking particularly of the Park Hospital, "the number and gravity of the surgical cases that come within the scope, demand for it the most careful and intelligent attention. Appreciating the importance of the position, the Board of Commissioners changed the jurisdiction of the Medical Board of Bellevue Hospital, and appointed one of its members as Surgeon-in-Chief, who, from his eminent talent, is well qualified for the position, but who, from the various distinguished posts he occupies and the extensive private practice he enjoys can not, from want of time, pay that constant supervision and personal attention to the Reception Hospitals as the interest of the public and the welfare required."

In reply, I wish to say that I was appointed to essentially the same office, which I now hold, in November, 1872, I believe before Mr. Stern was a Commissioner, and the reason assigned by the gentleman who called upon me to ask whether I would accept the position was, that great difficulty had been experienced in finding a surgeon to answer promptly the calls of resident physicians, and it was thought necessary to appoint one person who should hold himself responsible whenever called. I was assured that I would not be expected to make more than about one visit per week to the hospitals, (these hospitals are six miles apart), but that a summons would be expected to be answered promptly. I was also requested to suggest, from time to time, such alterations in their management as their condition might seem to me to demand.

At first my duties were supplementary to those of the Bellevue Surgeons, but subsequently the duty of acting as consulting and visiting Surgeon was devolved exclusively upon me.

I have already informed you how I have performed my duties as a consulting officer, having responded promptly to every summons. Before the appointment of Dr. Fluhrer to the place of Resident Physician at the Park Hospital, I was frequently summoned to this Hospital, and during my entire term of service a large portion of my visits to the 99th street Hospital were in response to calls. I have even denied myself my usual summer vacation in order that I might attend faithfully to this public duty.

Mr. Stern says that I performed no operations at the Park Hospital during the year of Dr. Fluhrers' service as Resident Physician and was present at but two, and he adds that the infrequency of my visits admits of but two explanations, either confidence in Dr. Fluhrer, or want of time. I will suggest another reason. During the year of Dr Fluhrer's service he has summoned me but three or four times, and most of the nine amputations as well as other capital operations made by him were made without my knowledge. I have more than once called his attention to this omission to summon me in these and other cases requiring capital operations, including a case of strangulated hernia, in some of which, at least, there was ample time for consultation. With one or two exceptions my presence during his operations was accidental. I made an amputation of the thigh on the 4th of April, and Dr. Fluhrer made one on the 21st, both patients died.

Mr. Stern compares the number of visits made by me to the Park Hospital during the year 1873, with those made by the Bellevue Hospital surgeons in 1872, and finds a difference of eighteen in favor of the latter, but he has omitted to make a similar comparison in relation to the 99th Street Hospital. Up to April 1, 1873, only eleven visits are recorded, and five of these were made by myself, while in 1873, I made fifty-one (51) visits to the 99th Street Hospital, and seventy-one (71) to thePark, in all, one hundred and twenty-two (122.)

The comparison of the results of operations in the two reception hospitals does great injustice to the able gentlemen who have been in charge of the 99th Street Hospital. Situated in a neighborhood where large public works are in progress, and where serious accidents from blasting, and the removal of heavy stones, are of frequent occurrence, the nature of the accidents has been constantly of the gravest character.

An examination of the records will show that eight amputations were made at the 99th Street Hospital, and nine at the Park. All of the amputations made at 99th street were in the lower extremities; 4 recovered and 4

died ; of those who died two (2) are reported in the books as having died of pyaemia, but I think one of them is recorded erroneously, and was probably septicæmia. No other cases of death from pyaemia are on record for that year. Of the nine (9) amputations at the Park Hospital, four (4) belonged to the upper extremities and five to the lower. Of the upper extremity amputations three out of four recovered ; of the lower extremity, two out of five (5) recovered. Surgeons understand that this difference as to fatality always exists between the two extremities, and the record in this point of view, as favorable for the 99th street than for the Park ; but in so small a number of cases this may be merely accidental. Certainly, the results furnish no evidence of the better management in the Park than in 99th street.

Mr. Stern says, pyaemia is stamped out at the Park, and that it continues a frequent cause of death at 99th street.

The records show one death from pyaemia at the Park, but as this was on the 7th day after Dr. Fluhrer took charge, he perhaps cannot be properly held responsible for the result ; and only two from the same cause at 99th street. But pyaemia is not peculiar to hospitals. Mr. Birket, of London, has recently reported twenty-three cases in private practice, and every surgeon meets with it occasionally, in the best ventilated and best constructed houses.

It is said in the communication referred to, that "there is another strong reason why a competent supervising surgeon should be engaged at the Reception Hospitals besides the present Surgeon-in Chief. The latter is one of the leading members of the Bellevue Medical Board, and it has often been insinuated that great prejudice exists in favor of Bellevue Hospital, and that thereby its members are liable to abuse the power of transfer of patients, and use the Reception Hospitals as feeders to clinics, transporting the greatest surgical injuries to a distant amphitheatre of operations "

Mr. Stern has never heard from any source such a charge made against me. I am conscious of a reputation above such a slander, and my years of faithful service under the Commissioners of Public Charities and Correction, without compensation, should have saved me so unjust an insinuation. So far as I know, during my term of service, the only instance in which a patient was sent to Bellevue for a clinic, was in the case of a patient with two broken thighs, sent to a Bellevue clinic by Dr. Fluhrer for the purpose of showing a mode of dressing devised by himself. This was done without my knowledge or approval.

Mr. Stern says: "The last nomination by the surgeon-in-chief of the Park Hospital staff was unfortunate, and was not done by concurrence, for all the appointments were made without examination, two of which have subsequently been rejected by an examining Board as incompetent to hold their positions." I do not understand what is meant by the statement that my nomination "was not done by concurrence," but as it is evidently intended to make me responsible for improper appointments, I reply that Mr. Stern knows that I first suggested and have constantly urged the necessity of competitive examinations and that your Board, and not myself, is responsible for their omission. I was required by you, however, to nominate, and I nominated the two gentlemen you mention, with others, all of whom were graduates in medicine. You decided subsequently that they should undergo an examination. Drs. Early and Campbell were examined, and recommended by the examining Board, and you appointed them. Of the two gentlemen said to have been rejected as incompetent by the Board, one did not come forward, as he was at the time on leave of absence in Kentucky. He was not therefore declared incompetent. The other had served at Charity Hospital, and as ambulance surgeon under your Board for more than a year, satisfactorily, and with his appointment I had nothing to do, but his advancement as a faithful officer I recommended when you insisted upon nominations. He was not declared by the examining Board (of which I was chairman) as incompetent to hold his position. He was subjected to a competitive examination for promotion, and failed to win. Nor is this surprising, when it is known that his competitors numbered about eighteen—that they were composed of cho ce men, and fresh from the three leading medical schools of the city, while the gentleman referred to had been more than a year out of his studies, and had but two days' notice that an examination would be required. It is no evidence of his lack of qualification or that he was incompetent for the place he occupied that he was distanced in a race so unequal. Such are the facts upon which Mr. Stern bases the statement that my nominations were "unfortunate."

You will pardon me, gentlemen, for having occupied your time with this long communication, but I was entrusted by you with certain duties which I have striven to perform in the spirit and letter of your wishes. And the character of Mr. Stern's communication seemed to render a full and complete statement of the facts relating to my administration necessary.

Very respectfully, yours,

FRANK H. HAMILTON,

Surgeon-in-Chief,

Reception Hospitals.

I wish to add that the number of cases of pyæmia during any one year either at the Ninety-ninth Street or Park Hospital has not been sufficient to imply that it was spread by infection, unless I except the year 1871, during which there were 4 cases in the Park Hospital. In 1870 there was 1 in the Park Hospital; in 1871 there were four; in 1872 there were 2, and in 1873 there were 1. Considering the large number of severe cases received during the several years it is quite probable they were all spontaneous and not due to infection. I have never seen any evidence that cases had originated from the latter source in either of the two hospitals.

